# Six-Month Trajectories of Symptoms, Perceived Recovery, and Functional Health in Ecuadorian Adults with COVID-19

**DOI:** 10.3390/jcm15207991

**Published:** 2026-10-15

**Authors:** Guido Mascialino, Alberto Rodríguez-Lorenzana, Trinidad Abiuso, Jack D. Watson, Paul B. Perrin, Juan Carlos Arango-Lasprilla, Freddy Palma, Cesar Parra-Gaete

**Affiliations:** 1 Health Sciences Department, Public University of Navarre, 31006 Pamplona, Spain; 2 Department of Veterans Affairs, Informatics, Decision Enhancement, and Analytic Sciences Center, Salt Lake City, UT 84108, USA; 3 Department of Internal Medicine, Division of Epidemiology, University of Utah School of Medicine, Salt Lake City, UT 84108, USA; 4 School of Data Science, Department of Psychology, University of Virginia, Charlottesville, VA 22903, USA; 5 Department of Cell Biology and Histology, University of the Basque Country, 48940 Leioa, Spain; 6 IKERBASQUE—Basque Foundation for Science, 48009 Bilbao, Spain; 7 Departamento de Salud y Seguridad Ocupacional, Universidad de Las Américas, Quito 170513, Ecuador; 8 Grupo de Investigación Bienestar, Salud y Sociedad, Escuela de Psicología y Educación, Universidad de Las Américas, Quito 170513, Ecuador

**Keywords:** post-acute COVID-19 syndrome, long COVID, health-related quality of life, Ecuador

## Abstract

**Background/Objectives**: Most longitudinal evidence on post-acute COVID-19 comes from hospitalized patients in high-income countries and less is known about community samples in low- and middle-income settings. This study examined changes in perceived recovery, symptom burden, general health perception, and functional difficulties over six months in Ecuadorian adults with confirmed COVID-19. **Methods**: In a prospective observational cohort in Quito, Ecuador, 120 adults with laboratory-confirmed COVID-19 were assessed at baseline (within four weeks of diagnosis) and again three and six months after the baseline assessment; 91 completed all three assessments. The Spanish-language ISARIC follow-up survey covered 39 symptoms, perceived recovery, the EQ-5D-5L and EQ-VAS, and six Washington Group functioning items rated retrospectively for the pre-COVID period and currently. Analyses were exploratory and descriptive, and all *p*-values are nominal and unadjusted for multiple comparisons. **Results**: Participants’ perceived complete recovery improved (χ^2^ = 15.24, *p* < 0.001), as did perceived general health (F(2, 178) = 5.80, *p* = 0.004). Chest pain, palpitations, muscle weakness, confusion, tinnitus, and diarrhea declined over follow-up. At six months, 37.4% still reported headaches, 35.2% fatigue, 35.2% confusion or inattention, and 25.3% sleep disturbance. Among EQ-5D-5L domains, only pain/discomfort improved (*p* = 0.012). Vision, walking, memory, and communication declined from pre-COVID to baseline and improved between baseline and six months; pre-COVID and six-month levels were not compared directly. **Conclusions**: Recovery over six months was gradual but incomplete, with a substantial minority reporting ongoing or recurrent symptoms at six months. These findings describe post-acute symptom burden following confirmed COVID-19 rather than formally diagnosed post-COVID-19 condition.

## 1. Introduction

COVID-19, caused by the SARS-CoV-2 coronavirus, ranges from asymptomatic infection to severe pneumonia, respiratory and cardiac failure, and sepsis [1,2]. The World Health Organization declared the outbreak a pandemic on 11 March 2020 [3], and early estimates placed the case fatality ratio at approximately 1.4%, rising sharply with age to about 13% in adults aged 80 years and older [4].

Although SARS-CoV-2 enters host cells primarily via the ACE2 receptor [5], it can also invade other organs, including the heart, kidneys, and intestines [6], aggravating the acute illness and contributing to longer-term consequences [7]. Neurological manifestations are common; one study reported them in 36.4% of the sample, including dizziness, headache, altered consciousness, stroke, and epilepsy [8]. Loss of taste and smell has also been reported [9] and has been attributed to viral access to the central nervous system through the olfactory route [10].

Symptoms continuing beyond four weeks from onset are commonly described as post-acute [11], whereas the World Health Organization consensus definition places post-COVID-19 condition at approximately three months from onset, with symptoms lasting at least two months and not explained by an alternative diagnosis [12]. Reported frequencies vary widely by setting; they occur in approximately 50–70% of hospitalized cases, 10–20% of non-hospitalized cases, and 10–12% of vaccinated cases [13], and the condition is best characterized in patients who have been hospitalized [14]. Frequently reported complaints include dyspnea, fatigue, cardiovascular problems, and joint and chest pain [15].

Ongoing symptoms can substantially affect cognitive and physical functioning [16], making quality of life a relevant outcome to evaluate in samples with COVID-19 [17].

One study [18] found that 32% of people with long COVID had severe functional limitations and needed help with many daily activities, while another study [19] reported that greater symptom severity was associated with worse overall health status. Consistent with these findings, a further study [20] linked a greater symptom burden after mild COVID-19 to poorer health status, poorer quality of life, and greater psychological distress, while a large community cohort study [21] found that 89% of participants had not returned to their pre-COVID level of health, with 38% reporting severely limited ability to work. Although the magnitude of these effects varies across populations and measurement approaches, the reported direction is consistent.

What remains less well characterized is how these outcomes evolve outside hospital settings and outside high-income countries. Longitudinal data from community-based, non-hospitalized cohorts in low- and middle-income settings, and from Latin America in particular, remain scarce. The present study therefore examined changes in perceived recovery, symptom burden, general health perception, and functional health difficulties over a six-month follow-up period in Ecuadorian adults with confirmed COVID-19, in order to provide descriptive evidence on post-acute symptom burden and functional recovery in a Latin American context.

## 2. Materials and Methods

### 2.1. Study Design and Setting

We conducted a prospective, longitudinal observational cohort study of adults with laboratory-confirmed COVID-19 in Quito, Ecuador. The study was carried out at Universidad de Las Américas (UDLA), with data collected over an 18-month period beginning in April 2022. Participants were assessed at three time points: baseline (T1) and again at 3 months (T2) and 6 months (T3). Baseline (T1) was defined as each participant’s first study assessment, completed within four weeks of their initial COVID-19 diagnosis; it thus captured symptom and functional status during the acute phase of illness. The T2 and T3 assessments were scheduled relative to each participant’s baseline visit rather than to the date of diagnosis or symptom onset. Because baseline could take place at any point within four weeks of diagnosis, the elapsed time from symptom onset to each assessment varied across participants, and the exact interval from onset or diagnosis to each assessment was not available for analysis. At the baseline assessment, participants additionally rated selected functional domains retrospectively for the pre-COVID period, providing a within-person reference point.

### 2.2. Participants

Adults who tested positive for COVID-19 were invited to participate by a community physician and by the physician serving the UDLA community at the time of their positive test. Eligible individuals were (a) 18 years of age or older, (b) diagnosed with COVID-19 confirmed by a documented positive RT-PCR or rapid antigen test result, (c) assessed at baseline within four weeks of their initial diagnosis, and (d) able to provide written informed consent. Individuals were excluded if they reported current alcohol or substance abuse or a pre-existing mental health condition. Both exclusion criteria were established by participant self-report at screening: a pre-existing mental health condition was operationalized as any self-reported psychiatric or psychological diagnosis predating COVID-19, and no structured diagnostic interview, chart review, or standardized screening instrument was used to verify it. Pre-existing chronic physical conditions were recorded but were not part of the exclusion criteria; the cohort therefore includes participants with physical comorbidity. Chronic conditions were ascertained through a single open-ended item in the sociodemographic questionnaire, which asked participants whether they had been diagnosed with any chronic medical condition that could affect their mental status or cognitive functioning. Responses were recorded as present or absent, and the specific conditions reported were not coded.

No formal a priori sample-size or power calculation was performed; as an exploratory, single-center descriptive study, the sample comprised all eligible adults recruited and retained over the enrollment period. A total of 120 adults were enrolled; 91 participants completed all three scheduled assessments and formed the longitudinal sample. Because of occasional missing responses to individual items, the sample size for specific analyses ranged from 88 to 91 participants (participant flow and sample characteristics are reported in the Results).

### 2.3. Measures

Sociodemographic questionnaire. An author-developed questionnaire collected age, sex, years of education, ethnicity, marital status, place of residence (rural or urban), employment status, and COVID-19 vaccination history.

ISARIC COVID-19 follow-up survey. Symptoms, health status, and functioning were assessed with the officially distributed Spanish-language version of the ISARIC Global COVID-19 Tier-1 follow-up survey (V1.2, 21 January 2021; CC BY-SA 4.0, University of Oxford). It comprised: (1) a symptom checklist of 39 symptoms across multiple organ systems, each reported as present or absent during the preceding seven days and framed as new or unusual since the onset of COVID-19; (2) items on perceived recovery and current health, including a single item on feeling completely recovered from COVID-19 and items on recent fever; (3) the EQ-5D-5L (mobility, self-care, usual activities, pain/discomfort, and anxiety/depression) plus the EQ-VAS (0–100 health “today”); and (4) six functioning items corresponding to the Washington Group Short Set on Functioning (seeing, hearing, walking, remembering or concentrating, self-care, and communicating), each on a four-level scale, rated both retrospectively for the pre-COVID period and for the current time.

### 2.4. Procedure

After providing informed consent, participants completed the baseline assessment in person at UDLA (within four weeks of diagnosis); the 3-month (T2) reassessment was administered by telephone, and the 6-month (T3) reassessment was again completed in person. Participants received a US$10 supermarket gift card. Each participant was assigned a code and data were stored without personal identifiers to protect confidentiality.

### 2.5. Ethical Considerations

The study was approved by the Comité de Ética de Investigación en Seres Humanos (CEISH) of the Pontificia Universidad Católica del Ecuador (approval no. CEISH-182-2021) and conducted in accordance with the Declaration of Helsinki. All participants provided written informed consent before participation.

### 2.6. Data Analysis Plan

All analyses were conducted in SPSS (version 31) [22]. Variables were analyzed according to their measurement type and study design. For binary symptom variables, differences across the three time points (baseline, 3 months, 6 months) were first assessed using Cochran’s Q test. For symptoms showing significant overall changes, McNemar’s tests were conducted to examine pairwise differences between baseline and 6 months, with exact *p*-values reported to account for small cell counts.

For the single continuous measure of general health (0–100 scale), a repeated-measures ANOVA was used to assess changes across all three time points. Where the overall effect of time was significant, a pairwise comparison between baseline and 6 months was performed with Bonferroni adjustment to evaluate sustained change.

Ordinal variables, including self-reported functional difficulties and other Likert-type items, were analyzed using the Friedman test to assess overall differences across time. Significant outcomes were followed by Wilcoxon signed-rank tests for pairwise comparisons, either between pre-COVID (retrospectively assessed) and baseline, or baseline and 6 months, depending on the variable’s structure and temporal relevance. For all analyses, descriptive statistics (frequencies and percentages for categorical/ordinal variables, mean ± SD for the single continuous variable) were reported, and statistical significance was set at α = 0.05.

Consistent with the study’s exploratory, descriptive aim, change across the three time points was examined for all outcomes, perceived recovery, individual symptoms, health-related quality of life (EQ-5D domains and EQ-VAS), and functional difficulties, which were treated as co-equal, exploratory outcomes. Because this was an exploratory, descriptive study, no correction for multiple comparisons was applied; individual symptom-level *p*-values are nominal and are interpreted as indicators of descriptive trends rather than confirmatory findings. Missing data were handled by complete-case analysis. No imputation was performed, and for each analysis participants missing the relevant variable at any assessment were excluded listwise, so that the analytic sample varied between 88 and 91 participants depending on the outcome.

## 3. Results

### 3.1. Participants Descriptive Statistics

A total of 120 Ecuadorian adults with confirmed COVID-19 participated in the study. Twenty-nine participants did not complete follow-up (10 at 3 months and 19 at 6 months) and were excluded from analyses. The remaining 91 participants completed all three scheduled assessments and formed the longitudinal sample. Because of occasional missing responses to individual items, the sample size for specific analyses ranged from 88 to 91 participants. For each analysis, participants missing the relevant variable at any assessment were excluded. The mean age of participants was 35.54 years (SD = 11.14), and 57.1% were female (Table 1). Participants included in and excluded from the longitudinal analyses were descriptively similar in age (M = 35.54, SD = 11.14 vs. M = 33.52, SD = 10.82), years of education (M = 18.86, SD = 3.35 vs. M = 18.66, SD = 3.75), and baseline symptom burden, with an average of 6.34 (SD = 5.25) and 6.50 (SD = 5.01) symptoms endorsed at baseline, respectively. Participants who did not complete follow-up were more frequently female (72.4% vs. 57.1%) and more frequently reported a chronic medical condition (31.0% vs. 15.4%). Both groups were fully vaccinated and comparable in residency, ethnicity, marital status, and work status (Table 1).

### 3.2. Perceived Recovery over Time

Participants’ self-reported feeling of complete recovery improved significantly over time (Friedman χ^2^ = 15.24, *p* < 0.001). Pairwise comparisons indicated a significant increase in recovery between baseline and 6 months (Wilcoxon *p* < 0.001). Despite this improvement, a subset of participants continued to report incomplete recovery at T3 (Table 2).

### 3.3. Symptom Burden over Time

Across the 39 symptoms assessed, several showed nominally significant improvement over time, including chest pain (Cochran’s Q *p* < 0.001; McNemar T1–T3 *p* < 0.001), palpitations (Q *p* < 0.001; McNemar *p* = 0.017), muscle weakness (Q *p* = 0.018; *p* = 0.002), confusion (Q *p* = 0.034; *p* = 0.015), ringing in the ears (Q *p* = 0.021; *p* = 0.018), and diarrhea (Q *p* = 0.039; *p* = 0.023). Several other gastrointestinal symptoms (loss of appetite and nausea/vomiting) showed differential movement over time and generally improving trends that did not reach significance in pairwise tests (Table 3). Because these comparisons were not adjusted for multiplicity, the *p*-values are nominal and exploratory and are best interpreted alongside the overall pattern of change in prevalence rather than individually.

Despite these improvements, a substantial proportion of participants continued to endorse ongoing or recurrent symptoms at 6 months. For example, 37.4% reported headaches, 35.2% reported fatigue, 35.2% reported confusion/lack of attention, and 25.3% reported sleep disturbances during the week preceding the T3 assessment (Table 3). Between 15–25% of participants also continued to report dyspnea, palpitations, tingling sensations, loss of appetite, nausea/vomiting, constipation, and stomach pain. Because symptom status was recorded separately at each assessment, these figures index the prevalence of symptoms reported at six months rather than continuous symptom persistence within individuals, and they may include ongoing, recurrent, or newly developed complaints. These symptoms were reported specifically as new or unusual since the onset of COVID-19, though they may also reflect unrelated conditions or seasonal illnesses.

### 3.4. General and Functional Health

Changes in general functional health were modest. Of the five domains assessed (mobility, self-care, usual activities, pain/discomfort, and anxiety/depression), only pain/discomfort showed significant improvement over time (Friedman χ^2^ = 8.82, *p* = 0.012; Wilcoxon T1–T3 *p* = 0.020; Table 4). Self-care showed a borderline overall effect (Friedman *p* = 0.050) that did not survive pairwise comparison (Wilcoxon T1–T3 *p* = 0.102); the remaining domains were statistically unchanged.

### 3.5. Health-Related Functional Difficulties

Participants retrospectively compared their pre-COVID functional abilities to their status at baseline. Significant declines were observed between pre-COVID and T1 for vision, walking, memory, and communication (all Wilcoxon *p* < 0.001; Table 5). Each of these domains also showed improvement between T1 and T3. Specifically, vision (*p* = 0.042), walking (*p* < 0.001), memory (*p* = 0.006), and communication (*p* = 0.024) all improved significantly between baseline and 6 months. Pre-COVID and 6-month ratings were not compared directly, so these analyses describe change relative to the acute phase rather than establishing whether functioning returned to pre-COVID levels; the distributions at each time point are shown in Table 5. Hearing and washing/self-care abilities did not differ significantly over time and had less than 16% and 3%, respectively, of the sample endorsing difficulties at any time point (Table 5).

### 3.6. General Health Perception

Perceived general health, rated on a continuous scale, improved significantly across the three time points (RM-ANOVA *F*(2, 178) = 5.80, *p* = 0.004, partial η^2^ = 0.061; Table 4). Post hoc tests indicated that perceived health was significantly higher at 6 months compared to baseline (*p* = 0.010; Table 4).

### 3.7. Summary of Trends

Overall, participants demonstrated gradual improvement in recovery status, general health, and select symptoms over the 6-month follow-up. Nevertheless, ongoing or recurrent symptoms, particularly fatigue, headache, sleep problems, gastrointestinal problems, and other neurological or sensory complaints, remained common at six months. Functional domains such as walking, remembering, and communicating showed measurable improvement relative to baseline, although difficulties were still reported by a subset of participants at 6 months; pre-COVID and 6-month levels were not compared directly.

## 4. Discussion

In this longitudinal cohort of Ecuadorian adults with confirmed COVID-19 followed at three time points (baseline, 3 months, and 6 months), we observed a pattern of gradual recovery alongside a substantial ongoing symptom burden. Perceived general health and the proportion endorsing complete recovery improved significantly by 6 months. Several acute symptoms, particularly chest pain, palpitations, muscle weakness, confusion/inattention, tinnitus, and diarrhea, declined over time. Nevertheless, a notable subgroup continued to report headaches (37%), fatigue (35%), confusion/inattention (35%), and sleep problems (25%) at 6 months. Functional domains showed modest but measurable gains: pain/discomfort improved, and retrospective comparisons suggested recovery in walking, memory, communication, and vision relative to the acute phase, although pre-COVID and 6-month functioning were not compared directly.

The co-occurrence of symptomatic improvement with continuing complaints underscores the heterogeneity of post-acute sequelae. The reporting of fatigue, headache, sleep disturbance, and attentional complaints at 6 months aligns with clinical narratives of long COVID characterized by fluctuating, multisystem symptoms. The improvement in pain/discomfort likely reflects gradual resolution of nociceptive and inflammatory contributors after acute illness [23], whereas continuing neurocognitive and sleep symptoms may indicate slower recovery trajectories in central mechanisms such as arousal and attention [24] and in interoceptive signaling [25]. Functionally, gains in mobility and communication reflect recovery from the acute phase; however, the difficulties still reported at six months, especially for memory and attention, highlight the potential impact on daily activities and work capacity, even in a relatively young, largely vaccinated sample.

An interpretive caveat concerns terminology. The ISARIC survey identifies symptoms described as new or unusual since COVID-19 at each assessment, but the present design did not verify that a given symptom was continuously present for at least two months in a given individual, nor did it systematically exclude alternative explanations. The cohort therefore describes post-acute symptom burden following confirmed COVID-19; it does not establish that individual participants met the formal clinical case definition of post-COVID-19 condition [12], and references to long COVID in this article should be read accordingly.

Broadly, these patterns are consistent with international cohort data reporting gradual symptomatic improvement over months, with a subset experiencing persistent fatigue, sleep disruption, and cognitive complaints [26,27]. The present study replicates and extends that literature by characterizing trajectories in an Ecuadorian sample and by pairing symptom trends with functional domains, thereby illustrating that perceived recovery and functional restoration can improve even while specific symptom clusters persist.

From a service-planning perspective, the findings are compatible with a stepped-care approach to post-COVID follow-up: (i) routine monitoring and education for most patients as symptoms gradually abate; (ii) targeted screening for fatigue, headache, sleep problems, and attentional complaints at 3–6 months; and (iii) timely referral to individualized rehabilitation for those with sustained limitations, including assessment for post-exertional symptom exacerbation and, when indicated, pacing or energy-management strategies, sleep hygiene interventions, and brief cognitive rehabilitation strategies [28]. None of these interventions was evaluated in the present cohort, and treatment exposure was not assessed; they are therefore presented as potential service implications informed by the broader literature rather than as outcomes demonstrated by the present results. The observed functional improvements suggest that many patients may experience meaningful recovery over time, while the ongoing symptom burden in a sizable minority argues for accessible outpatient pathways and, potentially, workplace accommodations.

### Limitations and Future Directions

Several limitations temper inference. First, complete-case analyses with listwise deletion, while pragmatic for an exploratory study, may bias estimates if missingness relates to symptom severity or recovery speed. Second, 29 of the 120 enrolled participants (24%) did not complete all three assessments. Participants who did not complete follow-up were comparable to completers in age, education, and baseline symptom burden, but were more often female and more often reported a chronic medical condition, so a degree of differential attrition cannot be excluded. Third, multiple hypothesis testing across many symptoms raises the risk of type I error; the study purposefully prioritized sensitivity to descriptive trends over familywise error control, so individual *p*-values are nominal and should be read as exploratory. Fourth, symptom and pre-COVID functional status were self-reported, introducing recall and reporting biases; the retrospective pre-COVID anchor is especially vulnerable to reconstruction effects. Fifth, follow-up assessments were scheduled relative to the baseline visit and the exact interval from symptom onset or diagnosis to each assessment was not available, so participants may have been at somewhat different phases of acute recovery when first assessed. Sixth, the sample was relatively small, young, entirely vaccinated, and highly selected (recruited at a single center through physician referral, with 92% urban residence and a mean of 18.9 years of education), which limits generalizability to older or unvaccinated populations and to less educated or rural communities. Seventh, the clinical characterization of the cohort is limited: acute COVID-19 severity, hospitalization during the acute illness, reinfection during follow-up, treatment exposure, and the timing of vaccination relative to infection were not recorded, and the specific chronic conditions reported by participants were not coded. These variables were therefore unavailable for the present analysis and could not be examined as potential modifiers of recovery. Eighth, the study did not include a SARS-CoV-2-naïve comparison group, so symptoms reported at six months cannot be attributed exclusively to COVID-19; some may reflect background morbidity, intercurrent or seasonal illness, or the broader circumstances of the pandemic period. Ninth, the 3-month assessment was administered by telephone whereas the baseline and 6-month assessments were completed in person, so mode of administration may have contributed to differences observed at that time point. Finally, the observational design precludes causal attributions regarding mechanisms of persistence or improvement.

Future research should (i) use missing-data-robust longitudinal models (e.g., generalized estimating equations or mixed-effects models) to leverage all available data; (ii) prespecify a limited set of primary endpoints or apply multiplicity adjustments; (iii) incorporate objective markers (e.g., neurocognitive testing, actigraphy for sleep, cardiopulmonary measures) to triangulate self-reports; (iv) extend follow-up beyond 6 months to characterize late recovery or chronicity; and (v) examine risk modifiers (e.g., age, comorbidities, initial severity) that may identify individuals needing early intervention. Studies should also record the exact interval from symptom onset or diagnosis to each assessment, state the permitted follow-up windows, and systematically characterize acute illness severity, hospitalization, reinfection, and treatment exposure so that these can be examined as modifiers of recovery. Embedding rehabilitation trials, particularly for fatigue management, sleep interventions, and cognitive rehabilitation, within longitudinal cohorts would directly inform care pathways.

## 5. Conclusions

Over 6 months, adults recovering from COVID-19 in this cohort demonstrated clear improvement in overall health, perceived recovery, and several acute symptoms, alongside ongoing or recurrent fatigue, headache, sleep disturbance, and attentional complaints in a substantial minority. Functional domains improved relative to the acute phase, although difficulties were still reported at six months. These descriptive data support proactive screening for lingering symptoms and are consistent with a gradual recovery trajectory for most patients in this cohort.

## Figures and Tables

**Table 1:** Sample characteristics of participants included in and excluded from the longitudinal analyses.

	Included (*n* = 91)		Excluded (*n* = 29)	
	*n* or M	% or SD	*n* or M	% or SD
Age (M, SD)	35.54	11.14	33.52	10.82
Years of Education (M, SD)	18.86	3.35	18.66	3.75
Sex (*n*, %)				
Male	39	42.90	7	24.10
Female	52	57.10	21	72.40
Residency (*n*, %)				
Rural	7	7.70	2	6.90
Urban	84	92.30	27	93.10
Ethnicity (*n*, %)				
Mestizo	85	93.40	27	93.10
Afro-Descendant	3	3.30	-	-
Caucasian	3	3.30	2	6.90
Marital Status (*n*, %)				
Single	50	54.90	17	58.60
Married/Common Law	36	39.60	11	37.90
Separated	2	2.20	1	3.40
Divorced	3	3.30	-	-
Work Status (*n*, %)				
Student	15	16.50	5	17.20
Full-Time	61	67.00	17	58.60
Part-Time	9	9.90	5	17.20
Freelance	4	4.40	-	-
Unemployed	1	1.10	2	6.90
Retired	1	1.10	-	-
Has Chronic Medical Condition (*n*, %)				
Yes	14	15.40	9	31.00
No	77	84.60	20	69.00
Received COVID Vaccine (*n*, %)				
Yes	91	100.00	29	100.00
No	-	-	-	-
Number of Vaccines/Boosters (*n*, %)				
1	1	1.10	-	-
2	10	11.00	5	17.20
3	69	75.80	21	72.40
4	11	12.10	3	10.30
Total Symptoms Endorsed at Baseline (M, SD)	6.34	5.25	6.50	5.01

**Table 2:** Current health related to COVID-19.

	Time 1		Time 2		Time 3		Cochran’s Q	Friedman	Wilcoxon T1–T3
	Frequency	Percent	Frequency	Percent	Frequency	Percent	*p*	*p*	*p*
Feeling of Complete Recovery							-	<0.001	<0.001
Completely Disagree	10	11.00	5	5.50	1	1.10			
Disagree	19	20.90	11	12.10	12	13.20			
Neither Agree nor Disagree	11	12.10	8	8.80	8	8.80			
Agree	24	26.40	21	23.10	24	26.40			
Completely Agree	25	27.50	45	49.50	46	50.50			
Missing/Unknown	2	2.20	1	1.10	-	-			
Felt Feverish Recently							0.943	-	-
No	85	93.40	84	92.30	84	92.30			
Yes	6	6.60	7	7.70	7	7.70			
Timeline of Fever							-	-	-
Did Not Report Fever Recently	85	93.40	84	92.30	84	92.30			
Last Week	1	1.10	1	1.10	4	4.40			
1–2 Weeks	1	1.10	5	5.50	1	1.10			
2–4 Weeks	3	3.30	1	1.10	1	1.10			
2–3 Months	-	-	-	-	1	1.10			
Missing/Unknown	1	1.10	-	-	-	-			
Cause of Most Recent Febrile Illness							-	-	-
COVID-19	3	3.30	1	1.10	-	-			
Other	3	3.30	5	5.50	7	7.70			

**Table 3:** Symptoms reported in the last seven days at each assessment.

Symptom	Time 1 *n* (%)	Time 2 *n* (%)	Time 3 *n* (%)	Cochran’s Q *p*	McNemar T1–T3 *p*
Headache	30 (33.0)	37 (40.7)	34 (37.4)	0.477	—
Persistent cough	21 (23.1)	13 (14.3)	12 (13.2)	0.088	—
Dyspnea	28 (30.8)	22 (24.2)	17 (18.7)	0.080	—
Chest pain	18 (19.8)	11 (12.1)	3 (3.3)	<0.001	<0.001
Palpitations	27 (29.7)	9 (9.9)	16 (17.6)	<0.001	0.017
Weight loss	13 (14.3)	5 (5.5)	10 (11.0)	0.066	—
Loss of appetite	19 (20.9)	7 (7.7)	16 (17.6)	0.009	0.097
Stomach pain	30 (33.0)	21 (23.1)	18 (19.8)	0.070	—
Nausea/vomiting	24 (26.4)	9 (9.9)	18 (19.8)	0.006	0.113
Constipation	11 (12.1)	12 (13.2)	17 (18.7)	0.244	—
Diarrhea	17 (18.7)	7 (7.7)	8 (8.8)	0.039	0.023
Menstruation changes	14 (15.4)	10 (11.0)	11 (12.1)	0.738	—
Swollen ankles	7 (7.7)	11 (12.1)	6 (6.6)	0.223	—
Balance problems	16 (17.6)	9 (9.9)	10 (11.0)	0.167	—
Muscle weakness	24 (26.4)	14 (15.4)	12 (13.2)	0.018	0.002
Fatigue	43 (47.3)	34 (37.4)	32 (35.2)	0.101	—
Muscle pain	14 (15.4)	8 (8.8)	11 (12.1)	0.276	—
Joint pain or swelling	13 (14.3)	11 (12.1)	9 (9.9)	0.580	—
Tingling sensation	20 (22.0)	15 (16.5)	19 (20.9)	0.549	—
Dizziness/lightheadedness	20 (22.0)	12 (13.2)	13 (14.3)	0.060	—
Weakness/fainting	12 (13.2)	6 (6.6)	6 (6.6)	0.120	—
Shivering/tremors	10 (11.0)	5 (5.5)	4 (4.4)	0.060	—
Confusion/lack of attention	44 (48.4)	34 (37.4)	32 (35.2)	0.034	0.015
Ringing in ears	25 (27.5)	17 (18.7)	13 (14.3)	0.021	0.018
Problems speaking/communicating	14 (15.4)	5 (5.5)	8 (8.8)	0.030	0.087
Sleeping problems	23 (25.3)	20 (22.0)	23 (25.3)	0.789	—
Skin rash	12 (13.2)	4 (4.4)	6 (6.6)	0.056	—

Note: For readability, the table presents the symptoms reported by at least 10% of participants at any assessment or showing a nominally significant change over time; results for the full set of 39 symptoms, including the complete response distributions, are provided in Supplementary Table S1. Percentages are calculated over the full longitudinal sample (*n* = 91). McNemar tests were computed only where the omnibus test was significant. Menstruation changes not applicable to all participants; percentages are nevertheless expressed over the full sample for comparability.

**Table 4:** Functional capacity.

							Friedman	Wilcoxon T1–T3	RM-ANOVA	Pairwise T1–T3
	Time 1		Time 2		Time 3		*p*	*p*	*p*	*p*
Mobility (*N*, %)							0.133	-	-	-
No Problems	79	86.80	85	93.40	87	95.60				
Mild Problems	10	11.00	5	5.50	2	2.20				
Moderate Problems	2	2.20	1	1.10	2	2.20				
Self-Care (*N*, %)							0.050	0.102		-
No Problems	88	96.70	91	100.00	91	100.00				
Mild Problems	2	2.20	0	0.00	0	0.00				
Moderate Problems	1	1.10	0	0.00	0	0.00				
Usual Activities (*N*, %)							0.078	-	-	-
No Problems	72	79.10	81	89.00	81	89.00				
Mild Problems	13	14.30	9	9.90	7	7.70				
Moderate Problems	5	5.50	1	1.10	3	3.30				
Could Not Do	1	1.10	0	0.00	0.00	0.00				
Pain/Discomfort (*N*, %)							0.012	0.020	-	-
None	51	56.00	67	73.60	67	73.60				
Mild	28	30.80	18	19.80	14	15.40				
Moderate	10	11.00	5	5.50	9	9.90				
Severe	2	2.20	1	1.10	1	1.10				
Anxiety/Depression (*N*, %)							0.155	-	-	-
Not At All	38	41.80	47	51.60	41	45.10				
Mild	29	31.90	27	29.70	34	37.40				
Moderate	19	20.90	14	15.40	11	12.10				
Very	5	5.50	2	2.20	4	4.40				
Extremely	-	-	1	1.10	1	1.10				
General Health (M, SD)	80.29	12.34	83.21	12.14	83.78	10.17	-		0.004	0.010

**Table 5:** Difficulties related to a health problem.

	Pre-COVID		Time 1		Time 2		Time 3		Wilcoxon Pre-COVID-T1	Wilcoxon T1–T3
	Frequency	Percent	Frequency	Percent	Frequency	Percent	Frequency	Percent	*p*	*p*
Vision									<0.001	0.042
No Difficulties	73	80.20	64	70.30	75	82.40	71	78.00		
Slightly Difficult	18	19.80	25	27.50	16	17.60	20	22.00		
Very Difficult	0	0.00	2	2.20	0	0.00	0	0.00		
Cannot Do at All	0	0.00	0	0.00	0	0.00	0	0.00		
Hearing									0.313	0.185
No Difficulties	78	85.70	79	86.80	85	93.40	76	83.50		
Slightly Difficult	12	13.20	11	12.10	5	5.50	14	15.40		
Very Difficult	0	0.00	0	0.00	1	1.10	0	0.00		
Cannot Do at All	0	0.00	0	0.00	0	0.00	0	0.00		
Missing/Unknown	1	1.10	1	1.10	-	-	1	1.10		
Walking									<0.001	<0.001
No Difficulties	81	89.00	66	72.50	72	79.10	81	89.00		
Slightly Difficult	10	11.00	24	26.40	18	19.80	7	7.70		
Very Difficult	0	0.00	1	1.10	1	1.10	2	2.20		
Cannot Do at All	0	0.00	0	0.00	0	0.00	0	0.00		
Missing/Unknown	-	-	-	-	-	-	1	1.10		
Remembering									<0.001	0.006
No Difficulties	61	67.00	39	42.90	54	59.30	46	50.50		
Slightly Difficult	28	30.80	42	46.20	31	34.10	39	42.90		
Very Difficult	2	2.20	10	11.00	6	6.60	5	5.50		
Cannot Do at All	0	0.00	0	0.00	0	0.00	0	0.00		
Missing/Unknown	-	-	-	-	-	-	1	1.10		
Washing									0.250	0.500
No Difficulties	91	100.00	89	97.80	89	97.80	89	97.80		
Slightly Difficult	0	0.00	2	2.20	1	1.10	2	2.20		
Very Difficult	0	0.00	0	0.00	0	0.00	0	0.00		
Cannot Do at All	0	0.00	0	0.00	0	0.00	0	0.00		
Missing/Unknown	-	-	-	-	1	1.10	-	-		
Communicating									<0.001	0.024
No Difficulties	75	82.40	65	71.40	82	90.10	73	80.20		
Slightly Difficult	15	16.50	23	25.30	7	7.70	16	17.60		
Very Difficult	1	1.10	3	3.30	1	1.10	2	2.20		
Cannot Do at All	0	0.00	0	0.00	0	0.00	0	0.00		
Missing/Unknown	-	-	-	-	1	1.10	-	-		

## Data Availability

The data presented in this study are available upon request from the corresponding author. The data are not publicly available because they contain information that could compromise the privacy of research participants.

## Supplementary Materials

The following supporting information can be downloaded at: https://www.mdpi.com/article/10.3390/jcm15207991/s1, Table S1: Symptoms experienced in the last seven days: full response distributions for all 39 symptoms assessed at each time point (*n* = 91). This table presents the complete version of Table 3 in the main text. *p*-values are nominal and unadjusted for multiple comparisons and should be interpreted as exploratory.
