# Supplementary material for: Six-Month Trajectories of Symptoms, Perceived Recovery, and Functional Health in Ecuadorian Adults with COVID-19
Source: J Clin Med. 2026 Oct 15;15(20):7991. doi: 10.3390/jcm15207991 (PMC13706103; doi:10.3390/jcm15207991)
Supplement: Supplementary file 1 [file jcm-15-07991-s001.zip › jcm-4495953-supplementary.pdf]

Supplementary Materials

Six-Month Trajectories of Symptoms, Perceived Recovery, and Functional Health in Ecuadorian Adults with COVID-19

Supplementary Table S1. Symptoms experienced in the last seven days: full response distributions for all 39 symptoms assessed at each time point (n = 91). This table presents the complete version of Table 3 in the main text. p-values are nominal and unadjusted for multiple comparisons and should be interpreted as exploratory.

	Time 1		Time 2		Time 3		Cochran's Q	McNemar T1-T3
	Frequency	Percent	Frequency	Percent	Frequency	Percent	*p*	Exact *p*
Headache							0.477	-
No	61	67.00	54	59.30	57	62.60		
Yes	30	33.00	37	40.70	34	37.40		
Persistent Cough							0.088	-
No	70	76.90	78	85.70	79	86.80		
Yes	21	23.10	13	14.30	12	13.20		
Smell Loss							0.420	-
No	82	90.10	85	93.40	85	93.40		
Yes	9	9.90	6	6.60	5	5.50		
Missing/Unknown	-	-	-	-	1	1.10		
Taste Loss							0.441	-
No	84	92.30	87	95.60	86	94.50		
Yes	7	7.70	4	4.40	4	4.40		
Missing/Unknown	-	-	-	-	1	1.10		
Dyspnea							0.080	-
No	63	69.20	69	75.80	74	81.30		
Yes	28	30.80	22	24.20	17	18.70		
Pain When Breathing							0.867	-
No	87	95.60	86	94.50	87	95.60		
Yes	4	4.40	5	5.50	4	4.40		
Chest Pain							< 0.001	< 0.001
No	73	80.20	80	87.90	88	96.70		
Yes	18	19.80	11	12.10	3	3.30		
Palpitations							< 0.001	0.017
No	64	70.30	82	90.10	75	82.40		

Yes	27	29.70	9	9.90	16	17.60		
Weight Loss							0.066	-
No	78	85.70	86	94.50	81	89.00		
Yes	13	14.30	5	5.50	10	11.00		
Loss of Appetite							0.009	0.097
No	72	79.10	84	92.30	75	82.40		
Yes	19	20.90	7	7.70	16	17.60		
Stomach Pain							0.070	-
No	61	67.00	70	76.90	73	80.20		
Yes	30	33.00	21	23.10	18	19.80		
Nausea/Vomiting							0.006	0.113
No	67	73.60	82	90.10	73	80.20		
Yes	24	26.40	9	9.90	18	19.80		
Constipation							0.244	-
No	80	87.90	79	86.80	74	81.30		
Yes	11	12.10	12	13.20	17	18.70		
Diarrhea							0.039	0.023
No	74	81.30	84	92.30	83	91.20		
Yes	17	18.70	7	7.70	8	8.80		
Problems Urinating							0.717	-
No	87	95.60	89	97.80	88	96.70		
Yes	4	4.40	2	2.20	3	3.30		
Erectile Dysfunction							0.549	-
No	40	44.00	41	45.10	36	39.60		
Yes	2	2.20	1	1.10	3	3.30		
Not Applicable	49	53.80	49	53.80	52	57.10		
Menstruation Changes							0.738	-
No	40	44.00	41	45.10	42	46.20		
Yes	14	15.40	10	11.00	11	12.10		
Not Applicable	37	40.70	40	44.00	38	41.80		
Swollen Ankles							0.223	-
No	84	92.30	80	87.90	85	93.40		
Yes	7	7.70	11	12.10	6	6.60		
Balance Problems							0.167	-
No	75	82.40	82	90.10	81	89.00		

Yes	16	17.60	9	9.90	10	11.00		
Muscle Weakness							0.018	0.002
No	67	73.60	77	84.60	79	86.80		
Yes	24	26.40	14	15.40	12	13.20		
Fatigue							0.101	-
No	48	52.70	57	62.60	59	64.80		
Yes	43	47.30	34	37.40	32	35.20		
Muscle Pain							0.276	-
No	77	84.60	83	91.20	80	87.90		
Yes	14	15.40	8	8.80	11	12.10		
Joint Pain or Swelling							0.580	-
No	78	85.70	80	87.90	82	90.10		
Yes	13	14.30	11	12.10	9	9.90		
Inability to Move or Control Movement							0.607	-
No	90	98.90	90	98.90	91	100.00		
Yes	1	1.10	1	1.10	0	0.00		
Numbness on One Side of Face or Body							0.717	-
No	90	98.90	90	98.90	89	97.80		
Yes	1	1.10	1	1.10	2	2.20		
Tingling Sensation							0.549	-
No	71	78.00	76	83.50	72	79.10		
Yes	20	22.00	15	16.50	19	20.90		
Dizziness/Lightheadedness							0.060	-
No	70	76.90	79	86.80	78	85.70		
Yes	20	22.00	12	13.20	13	14.30		
Missing/Unknown	1	1.10	-	-	-	-		
Weakness/Fainting							0.120	-
No	79	86.80	85	93.40	85	93.40		
Yes	12	13.20	6	6.60	6	6.60		
Seizures							-	-
No	91	100.00	91	100.00	91	100.00		
Yes	0	0.00	0	0.00	0	0.00		
Shivering/Tremors							0.060	-

No	81	89.00	86	94.50	87	95.60		
Yes	10	11.00	5	5.50	4	4.40		
Confusion/Lack of Attention							0.034	0.015
No	46	50.50	57	62.60	59	64.80		
Yes	44	48.40	34	37.40	32	35.20		
Missing/Unknown	1	1.10	-	-	-	-		
Swallowing or Chewing							1.000	-
No	88	96.70	88	96.70	88	96.70		
Yes	3	3.30	3	3.30	3	3.30		
Vision Problems							0.846	-
No	83	91.20	82	90.10	84	92.30		
Yes	8	8.80	9	9.90	7	7.70		
Ringing in Ears							0.021	0.018
No	66	72.50	74	81.30	78	85.70		
Yes	25	27.50	17	18.70	13	14.30		
Problems Speaking/Communicating							0.030	0.087
No	77	84.60	86	94.50	83	91.20		
Yes	14	15.40	5	5.50	8	8.80		
Sleeping Problems							0.789	-
No	68	74.70	71	78.00	68	74.70		
Yes	23	25.30	20	22.00	23	25.30		
Bumps/Rashes							0.779	-
No	88	96.70	89	97.80	90	98.90		
Yes	2	2.20	1	1.10	1	1.10		
Missing/Unknown	1	1.10	1	1.10	-	-		
Skin Rash							0.056	-
No	79	86.80	87	95.60	85	93.40		
Yes	12	13.20	4	4.40	6	6.60		
Hemorrhage							0.246	-
No	90	98.90	88	96.70	89	97.80		
Yes	1	1.10	3	3.30	2	2.20		
